# Electromyography-Guided Adjustment of an Occlusal Appliance: Effect on Pain Perceptions Related with Temporomandibular Disorders. A Controlled Clinical Study

**DOI:** 10.3390/diagnostics11040667

**Published:** 2021-04-08

**Authors:** Simona Tecco, Vincenzo Quinzi, Alessandro Nota, Alessandro Giovannozzi, Maria Rosaria Abed, Giuseppe Marzo

**Affiliations:** 1 Faculty of Medicine, Vita-Salute San Raffaele University, I.R.C.C.S. San Raffaele Hospital, 20132 Milano, Italy; nota.alessandro@hsr.it; 2 Department of Life, Health and Environmental Sciences, University of L’Aquila, 67100 L’Aquila, Italy; vincenzo.quinzi@univaq.it (V.Q.); giuseppe.marzo@cc.univaq.it (G.M.); 3 Private Practice, 00040 Roma, Italy; alegiovannozzi@gmail.com (A.G.); simtecc@tin.it (M.R.A.)

**Keywords:** occlusal appliance, electromyography, temporomandibular joint disorders, muscle pain, removable appliance

## Abstract

Background: The purpose of this study is to evaluate the effect of an electromyography-guided adjustment of an occlusal appliance on the management of Temporomandibular disorder-related pain. Methods: Data from 40 adult patients (20 males and 20 females), who underwent treatment with occlusal appliances, were recorded. A total of 20 appliances were adjusted according to electromyographic data (group 1), while the others were adjusted by a clinical conventional procedure (group 2). Muscle pain to palpation, pain during articular movements and headache were recorded by a VAS score (from 0 to 100) before the beginning of treatment (T0), at T1 (4 weeks) and T2 (8 weeks). Results: Results showed a reduction of pain in both groups, with a better trend for group 1, where better results were achieved at T1 and maintained stability at T2, with an improved mean value regarding all parameters studied. After 8 weeks, only small recurrences started to occur in muscle pain to palpation in group 2. Conclusions: An occlusal appliance seems to be able to achieve a clinical improvement of Temporomandibular disorder (TMD)-related pain and headache, independently from the adjustment procedure adopted. However, the use of a surface electromyographic activity of masticatory muscles (sEMG) device as an aid in the calibration procedure seems to allow a better trend because the improvement of symptoms was obtained before, after the first four weeks, with an improvement in percentages of all the variables investigated. While the conventional procedure obtained later the improvement.

## 1. Introduction

Temporomandibular disorders (TMDs) are a heterogeneous group of clinical dysfunctions involving the masticatory muscles and/or temporomandibular joints (TMJ) and associated structures (American Association of Dental research. Policy Statement on TDM. March 2010—reaffirmed 2015—http://www.iadr.org/AADR/About-Us/Policy-Steatments/Science-Policy/Temporomandibular-Disorders-TMD, accessed on 1 February 2021). TMDs are the most prevalent orofacial pain condition, among inflammation (e.g., sinusitis), vascular compression (e.g., vascular migraines), other disorders of the musculoskeletal, neurological and/or neuropathic involvement (e.g., trigeminal neuralgia), and idiopathic trigeminal pain [1].

In general, TMD is believed to affect anywhere between 5 and 15% of adults in the population [2]. Interestingly, there is evidence that the prevalence of TMD appears to be increased in recent years [2].

TMDs are diffused in males and females with a prevalence of female gender, and are also observed in the pediatric and adolescent population (about 11% was reported) [2].

To date, the main guidelines on TMDs management are provided by the American Association of Dental Research (AADR), which read verbatim as follows: “The signs and symptoms associated with these disorders are diverse, and may include difficulties with chewing, speaking, and other orofacial functions. They also are frequently associated with acute or persistent pain, and the patients often suffer from other painful disorders (comorbidities)”. 

TMDs are classified from painless clicking of the joint (Stage I) to severe degenerative bony changes (Stage V) [2]. In some cases, a patient is diagnosed with multiple diagnoses, and often those diagnoses may change as the disease progresses or resolves.

Chewing problems include intra-articular sounds, reduced range of motion of the lower jaw, pain and discomfort pressing the area around TMJ, or masticatory muscles.

Some signs and symptoms resolve spontaneously even without treatment, whereas others persist for years despite all treatment options having been exhausted [2].

Treatments include the use of occlusal appliances, sometimes surgical procedures as arthrocentesis, cognitive behavioral therapy for muscle parafunction, and other various treatments involving other specialists (physiotherapy, for example) [2].

Again, today, occlusal appliances are the most widely used intraoral devices for the management of pain, due to the reversibility of the procedure [3]. The desired outcomes of reversible therapy with occlusal appliances are essentially a reduction in the Algic component, masticatory muscle relaxation and, hopefully, reduction of headache [4]. Therefore, TMDs are also associated with the presence of intra-articular sounds (clicks) and occlusal splint often reduces their frequency because of its capability to re-establish immediately the normal condyle/disk relationship [2,5].

Therefore, the success of reversible therapy on pain appears to be paramount for long-term rehabilitation [6]. According to the literature, however, there is a lack of clarity regarding the management of occlusal appliances by the clinicians, due to different existing protocols for their adjustment. For example, Wiens (2016) has shown the technical advances over time, but did not reflect a desired clinical outcome [7]. An optimum adjustment should include point-like homogeneous contact points on the appliance, all distributed on the dental arch. The clinical effect of the occlusal appliance should be an improvement of signs and symptoms. 

Undoubtedly, it is worth mentioning that the clinical effects of occlusal appliances for the different types of disorders may suffer from the role played by the practitioner itself during the clinical conventional procedure of its adjustment [7,8].

One of the emerging digital procedures for the adjustment of intraoral appliances or prostheses is based on the analysis of surface electromyographic activity of masticatory muscles (sEMG), which monitor the synergistic action of muscles in order to evaluate their balanced function [8,9]. This approach is based on data that highlight how symmetry in the electromyographic activity of the masticatory muscles is necessary for oral [9,10] as well as functional rehabilitation [11]. However, on these digitized procedures, there are no studies that have evaluated the results from a clinical and symptomatological point of view (on pain).

Thus, the purpose of this observational study was to analyze the effects of an electromyography-guided adjustment of an occlusal appliance on muscular pain comparing it with a standard procedure in a control group.

## 2. Materials and Methods

**Figure 1:**
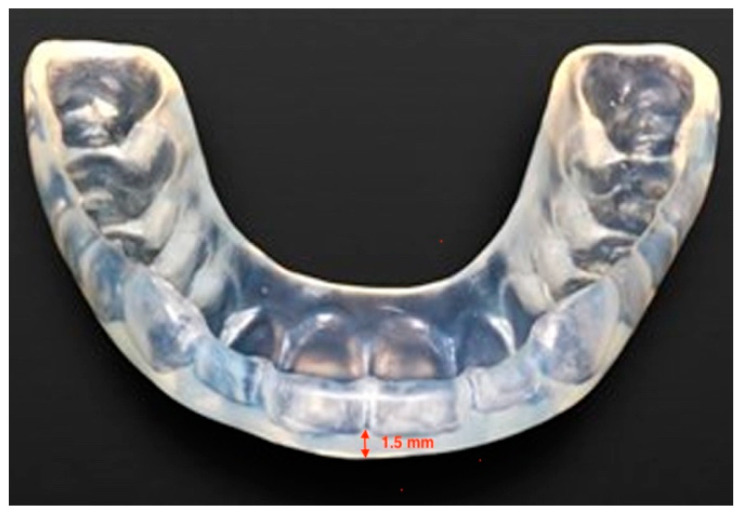
Individualized 1.5 mm thick occlusal appliance, made of a heat-cured acrylic resin (Duran^®^, Scheu-Dental Technology, Iserlohn, Germany).

The present observational protocol was approved by the Ethics Committee of the University of L’Aquila, Italy (Document DR206/2013, dated 10 January 2014). Data from a sample of 40 subjects, 20 males and 20 females, aged between 20 and 30 years old (average 25 years), who were going to receive an occlusal appliance for the management of TMD at George Eastman Dental Hospital in Rome (Italy) were selected for the present study. All the patients complained of muscle tension headache, associated with masticatory muscle pain to palpation, as well as pain during mandibular movements. None of them was affected by disc displacement, or degenerative joint disease. All patients were treated with an individualized 1.5 mm thick occlusal appliance, made of a heat-cured acrylic resin (Duran^®^, Scheu-Dental Technology, Iserlohn, Germany), applied in their lower jaw (Figure 1). 

In the study group (the study group) (20 patients) the calibration was performed by achieving a condition of muscular balance and relaxation according to sEMG data, whereas in the control group (the control group) (20 patients) it was performed by a standard procedure aimed to achieve homogeneous point-shaped dental contacts on the appliance [12]. The patients were instructed to wear the appliance 22 h per day, removing it only for meals and were treated by the same expert operator (the author A.G.) In the study group, sEMG was performed using TEETHAN^®^ (Teethan S.p.a. Garbagnate Milanese, Milan, Italy) a 4-channel wireless electromyograph, featuring surface electrodes placed at the level of the masseter muscles and anterior temporalis muscle [13,14]. All patients underwent clinical follow up during the subsequent weeks, and painful muscles at palpation, pain arising during mandibular function (functional pain) and headache, were recorded by using the VAS scale, on a range of scores from 0 to 100. Follow-ups were scheduled after 4 weeks (T1) and after 8 weeks (T2) from in the initial phase.

### Data Handling and Statistical Analyses

The sample size was evaluated a priori by performing analysis for estimating the minimum number of subjects to achieve a statistical power of 80% with alpha 0.05 on the comparison between groups for the primary outcome. The results showed that a minimum of 18 subjects per group was required. Applying the Shapiro–Wilk W test normal distribution of data was confirmed for the variables muscular pain at palpation (Shapiro–Wilk W = 0.95; *p* = 0.14) and headache (Shapiro–Wilk W = 0.96; *p* = 0.19). Differently, for functional complaints, data did not show a normal distribution (Shapiro–Wilk W = 0.92; *p* = 0.008). Descriptive statistics for the variables muscular pain at palpation and headache included mean and standard deviation. While functional pain was described as median with 25th and 75th percentiles. In order to analyze the variables of muscular pain at palpation and headache, a *t*-test for independent samples was performed to analyze the differences between the groups at each time point while a one-way ANOVA test was adopted to analyze the significance of changes over time. When significant, a post-hoc Tukey test was employed to further illuminate the statistically significant differences. For the variable of functional pain, the Friedman test and the Wilcoxon signed rank test were used to evaluate intra-groups differences; while the Mann–Whitney test was adopted to evaluate between groups differences at T0, T1 and T2. Statistical analyses were performed with the software StatPlus Pro for MAC (build 7.3.3.0/Core v7.3.32; AnalystSoft Inc., 2020, Walnut, CA, USA). For each test, p was set at 0.05 level. 

## 3. Results

Table 1 reports descriptive statistics for the variables muscular pain at palpation and headache. Table 2 reports descriptive statistics for the variable functional pain.

### 3.1. Muscular Pain at Palpation

**Figure 2:**
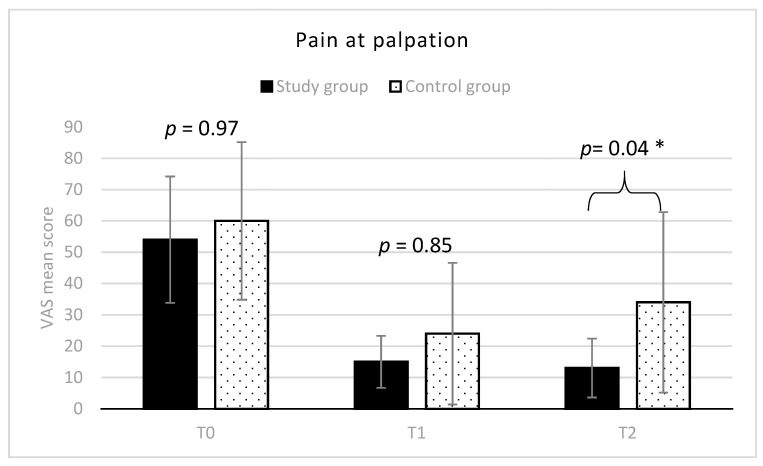
Muscle pain at palpation (mean VAS scores and SD) in the two groups. * indicates between groups statistically significant differences (*p* < 0.05).

At T0, VAS score averaged 54 in group 1 and 60 in group 2 (range: 10–90 for the whole sample), without any statistically significant difference between the two groups. While at T2, a significantly lower mean VAS score was observed in the study group respect to the control group (mean difference = −21; 95% C.I. = −43.04–1.04; t = 3.22; *p* < 0.05) (Figure 2).

**Figure 3:**
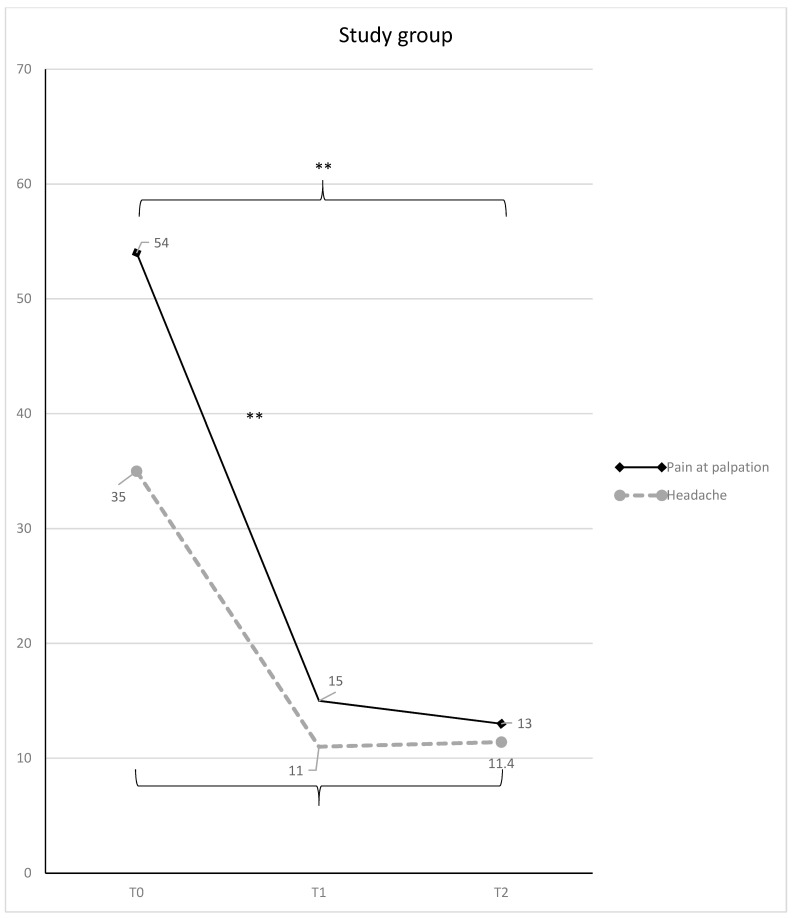
Muscle pain at palpation and headache (mean VAS scores and SD) in the study group, with statistically significant differences overtime (* = *p* < 0.05; ** = *p* < 0.01).

Considering the trend of the variable in each group over time, VAS significantly decreased at T1 in the test group, achieving a mean value of 15 (T0-T1 mean difference = 39; 95% C.I. = 16.95–61.04; t = 5.99; *p* < 0.01), and it remained almost stable at T2, with a mean value of 13 (T0-T2 mean difference = 41; 95% C.I. = 18.95–63.04; t = 6.29; *p* < 0.01) (Figure 3). 

**Figure 4:**
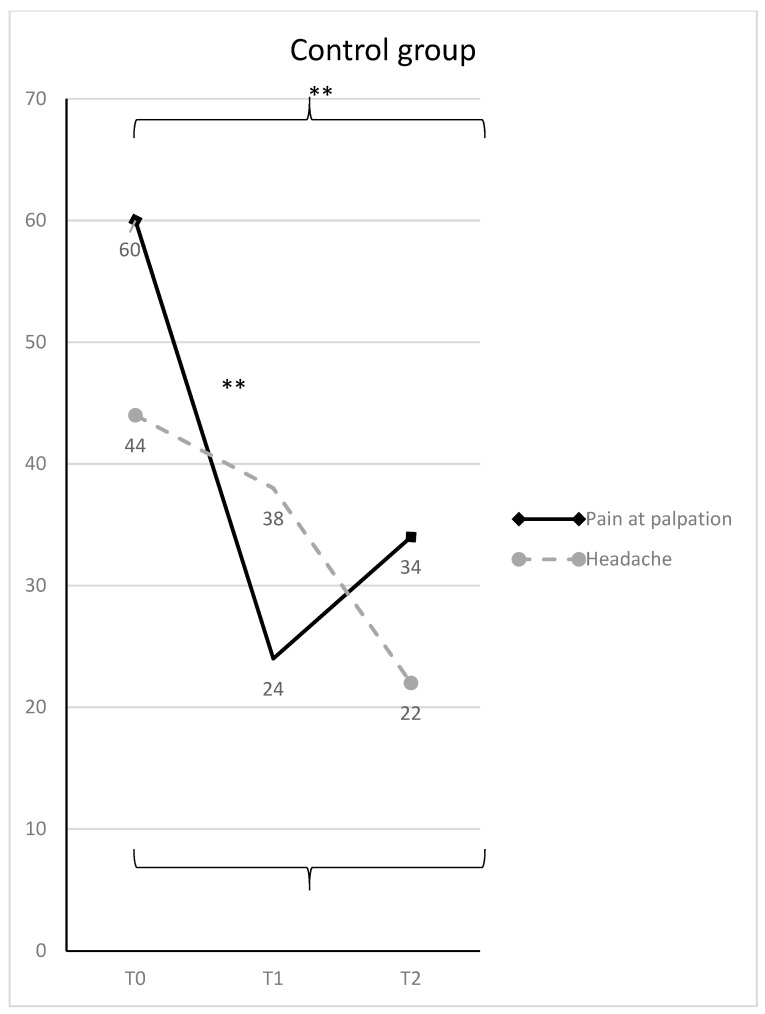
Muscle pain at palpation and headache (mean VAS scores and SD) in the control group, with statistically significant differences overtime (* = *p* < 0.05; ** = *p* < 0.01).

In the control group, it scored from 60 to 24 at T1 (T0-T1 mean difference = 36; 95% C.I. = 13.95–58.04; t = 5.53; *p* < 0.01), and 34 at T2 (T0-T2 mean difference = 26; 95% C.I. = 3.95–48.04; t = 3.99; *p* < 0.01) (Figure 4).

### 3.2. Functional Pain

**Figure 5:**
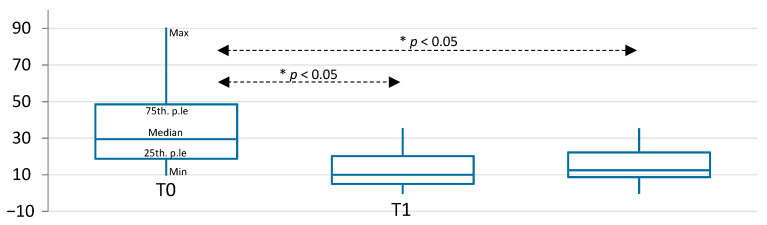
Box plots represent median, 25th and 75th percentiles of VAS scores for functional pain in the study group over time. * indicates statistically significant differences, *p* < 0.05.

At T0, the median VAS score in the study group 29.5, and it was 52.5 in the control group, (range: 10–90 for the whole sample), without any statistically significant difference between the two groups. At T1 the study group showed a statistically significant lower VAS score, with respect to the control group (Mann–Whitney U = 282,000; *p* = 0.026) (Table 2). No statistically significant differences were observed at T2. Considering the trend over time, the study group experimented with a statistically significant reduction of pain over time, from T0 to T1 (Wilcoxon ranks z = −3.82; *p* < 0.01); and from T0 to T2 (Wilcoxon ranks z = −3.57; *p* < 0.01) (Figure 5). 

**Figure 6:**
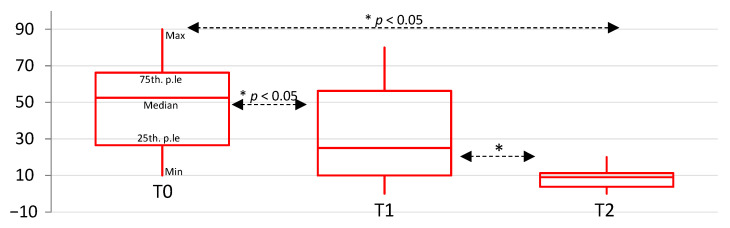
Box plots represent median, 25th and 75th percentiles of VAS scores for functional pain in the control group over time. * indicates statistically significant differences, *p* < 0.05.

The control group showed a statistically significant decrease of VAS score from T0 to T1 (Wilcoxon ranks z = −3.40; *p* < 0.01); from T0 to T2 (Wilcoxon ranks z = −3.74; *p* < 0.01); and from T1 to T2 (Wilcoxon ranks z = −3.04; *p* < 0.01) (Figure 6).

### 3.3. Muscular Tension Headache

**Figure 7:**
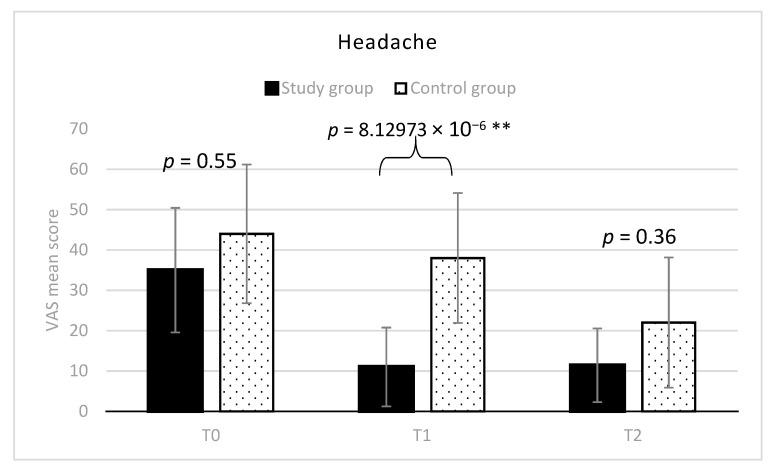
Headache at palpation (mean VAS scores and SD) in the two groups. ** indicates between groups statistically significant differences (*p* < 0.01).

The trend of headache VAS score for both the groups, at any stage, is depicted in Figure 7. 

At T0, VAS averaged 35 in group 1, and 44 in group 2 (range: 10–70 for the whole sample) without any statistically significant difference between the two groups. At T1, the study group showed a significantly lower mean VAS score, respect to the control group (mean difference = 27; 95% C.I. = 42.34–11.65; t = 5.95; *p* < 0.01). However, there was not any statistically significant difference between the two groups at T2. Considering the trend over time, VAS significantly decreased in the study group till a value of 11 (mean difference T0-T1 = 24; 95% C.I. = 8.65–39.34; t = 5.29; *p* = 0.0001) and remained almost stable at T2 (mean difference T0-T2 = 23; 95% C.I. = 8.25–38.94; t = 5.20; *p* = 0.0001) (Figure 3). Differently, in the control group VAS scores decreased overtime more slowly and became 38 at T1, and 22 at T2 (T0-T2 mean difference = 22; 95% C.I. = 6.65–37.34; t = 4.85; *p* = 0.00059) (Figure 4). The percentages of VAS score improvement for all the considered variables in the two groups are reported for both the groups in Table 3.

## 4. Discussion

This observational study was aimed to compare the effect of occlusal appliances adjusted with sEMG aid (the study group) versus the conventional adjustment procedure (the control group) in the management of TMDs related pain. 

The comparison of the VAS scores between the two groups showed some improvements for both groups. However, it seems that there was a better trend for the study group, respect to the control group. For the study group, the improvement of symptoms was obtained after the first four weeks (T0-T1 difference), with an improvement in percentages of all the variables investigated. While the control group showed a slightly different trend after the first four weeks of treatment, with a lower improvement (in percentage) than the study group. In addition, the control subjects showed a recurrence of light symptoms as shown by the score obtained for muscle palpation after the first four weeks, between T1 and T2, but results indicate that there was a statistically significant improvement of this variable from T0 to T2. Overall, in conclusion, between T1 and T2 there were positive results, for both the groups, according to all the other variables.

Thus, the present observations confirm that the most common and widespread therapy procedure for TMDs consisting of the use of occlusal appliances, is useful in controlling the pain related to altered muscular activity [7,8]. Thus, the present findings seem to support the principle that occlusal appliances could be able to maintain a primary role in the symptomatic treatment of TMD patients, allowing a change in the distribution of joint load vectors and relaxation of muscle fibers. 

The TMJ is located near a major nerve in the face, which is at the center of a network of nerves that connect throughout the face, head and neck. So when the TMJ is affected, pain can spread throughout the face, head and neck (the eyes, ears, mouth, forehead, cheeks, tongue, teeth and throat). Even the muscles of the neck can be involved.

TMDs are diffused in males and females with a prevalence of female gender [15]. They are also observed in the pediatric and adolescent population, (about 11% was reported) [2] in which they were related also to poor cervical posture, [16] and in some cases occlusal appliances were also referend to influence the general mandibular posture [17]. Diagnosis is made through an anamnestic questionnaire and clinical exam with palpation [18].

For the study group, the best results were obtained after the first four weeks (T0-T1 difference), with an improvement in percentages of all the variables investigate: in particular, muscular pain at palpation improved by 72.2%, headache by 68.5% and functional pain by 66.6%, with statistically significant differences. The control group showed a slightly different trend after the first four weeks of treatment, with a lower improvement (in percentage) than the study group: muscular pain at palpation improved by 60%, headache by 13% and functional pain by 36%. In the control group, headache showed an improvement later than other symptoms, as results indicate that there was a statistically significant improvement of this variable from T0 to T2 in group 2. After the first four weeks, between T1 and T2, there was a recurrence of light symptoms as shown by the score obtained by the control group for muscle palpation (41% worsening between T1 and T2) (Figure 4). Overall, between T1 and T2, there were positive results for both the groups, according to all the other variables. It can be concluded from the present data that the general outcome was an overall improvement for both groups between T0 and T2. 

The results observed in the study group, where the occlusal appliance adjustment was aided by sEMG, seem to suggest that a better adjustment of the appliance was performed, helping the clinician to increase the predictability in the balance of the bilateral contacts of the occlusal appliance, as previously suggested [19,20,21,22]. The use of electromyography to adjust an occlusal splint is only one of the techniques that can be used for the adjustment of an occlusal splint, so the present results cannot be generalized for all the other technique. 

Multiple designs are available, such as hard, soft, and anterior repositioning splint. At present, there is no consensus on which design is superior, as results from different studies are equivocal in terms of the efficacy of different designs of occlusal splints [2].

It should be considered that the traditional procedure applied in the control group, without using any digital equipment, could bring results more dependent on the practitioner’s expertise. The worsening percentage for muscle pain to palpation, registered between T1 and T2 in group 2 may be justified by the fact that, after an initial unlocking of the occlusion and subsequent improvement of the symptomatology, the modified occlusion could have determined the onset of new symptoms. For this reason, occlusal appliances are generally preferred and recommended against irreversible treatments, as modifying the occlusion in the long term could expose the patient to the risk of recurrence of symptomatology. On the other hand, the sEMG seems a useful method for improving the quality and predictability of the appliance adjustment, even though it also requires a learning curve for its use.

## 5. Conclusions

An occlusal appliance seems to be able to achieve a clinical improvement of TMDs related pain and headache, independently from the adjustment procedure adopted. 

However, the use of an sEMG device as an aid in the calibration procedure seems to allow a better trend because the improvement of symptoms was obtained before and after the first four weeks, with an improvement in percentages of all the variables investigated. Meanwhile, the conventional procedure was obtained later than the improvement.

Future studies will clarify the effects of other material-based appliances or adjustment procedures for clinics.

## Figures and Tables

**Table 1:** Descriptive statistic for muscular pain at palpation and headache. VAS scores mean ± standard deviation (SD).

	Test Group			Control Group		
	T0 Mean ± SD	T1 Mean ± SD	T2 Mean ± SD	T0 Mean ± SD	T1 Mean ± SD	T2 Mean ± SD
VAS (Muscular Pain at Palpation)	54 ± 20.2	15 ± 8.29	13 ± 9.36	60 ± 25.17	24 ± 22.61	34 ± 28.82
VAS (Headache)	35 ± 15.44	11 ± 9.76	11.4 ± 9.13	44 ± 17.18	38 ± 16.08	22 ± 16.13

**Table 2:** Descriptive statistic for functional pain. VAS scores, median, 25th and 75th percentiles.

	Test Group			Control Group		
	T0 Median (25th and 75th p.le)	T1 Median (25th and 75th p.le)	T2 Median (25th and 75th p.le)	T0 Median (25th and 75th p.le)	T1 Median (25th and 75th p.le)	T2 Median (25th and 75th p.le)
VAS (Functional Pain)	29.5 (18.75–48.5)	10 * (5–20.25)	12.5 (8.75–22.25)	52.5 (26.5–66.25)	25 (10–56.25)	9 (3.75–11.25)

* *p* < 0.05.

**Table 3:** Intra-group differences expressed in percentage for both the groups.

	Group 1			Group 2		
	T0-T1	T1-T2	T0-T2	T0-T1	T1-T2	T0-T2
Muscular Pain at Palpation	−72.22%	−13.32%	−75.92%	−60.00%	+41.66%	−43.33%
Headache	−68.57%	+3.63%	−67.42%	−13.63%	−42.10%	−50%
Functional Pain	−66.66%	+25%	−58.33%	−36.00%	−62.5%	−76%

## Data Availability

The data that support the findings of this study are available from the University of L’Aquila, but restrictions apply to the availability of these data, which were used under license for the current study, and so are not publicly available. Data are however available from the authors upon reasonable request and with permission of the University of L’Aquila partner.
